# Reflections on Leadership: Lessons from the APSI — Dr. S Raja Sabapathy Leadership Award (Supported by Dr. Somes Guha)

**DOI:** 10.1055/s-0045-1810009

**Published:** 2025-09-01

**Authors:** Nikhil Panse

**Affiliations:** 1 Department of Plastic Surgery, Byramjee Jeejeebhoy Government Medical College, Pune, India

**Keywords:** leadership award, Association of Plastic Surgeons of India, BETiC, health care innovation, leaders in plastic surgery

## Abstract

The APSI—Dr. S Raja Sabapathy Leadership Award (supported by Dr. Somes Guha) aims to recognize and nurture emerging leaders in Indian plastic surgery. This reflective piece captures my experience as the fourth recipient of the award in the year 2022, my visit to BETiC (Biomedical Engineering and Technology Incubation Centre) at IIT Bombay, and the leadership lessons gleaned from the experience. It emphasizes the significance of daily decision-making and creating a culture of empowerment. The reflection also underscores how interdisciplinary learnings can strengthen leadership practices in clinical settings and foster innovation, collaboration, and growth. This brief communication is intended to inspire young plastic surgeons to embrace leadership as a continuous journey.

## Introduction

The APSI—Dr. S Raja Sabapathy Leadership Award (supported by Dr. Somes Guha) aims to recognize and nurture emerging leaders in Indian plastic surgery. 1 This reflective piece captures my experience as the fourth recipient of the award, my visit to BETiC (Biomedical Engineering and Technology Incubation Centre) at IIT Bombay, and the leadership lessons gleaned from the experience.

Leadership is often seen through the lens of titles and accolades. Yet, in the day-to-day reality of running a department in a government hospital, or in a corporate set up or even one's own private practice, leadership is a practice grounded in consistency, empathy, and vision. Receiving the APSI—Dr. S Raja Sabapathy Leadership Award in 2023 was not just an honor but also compelled me to reflect on my journey and evaluate what effective leadership truly means in a resource-limited yet potential-rich environment, and our day-to-day lives. This brief writeup reflects the lessons learnt, and its impact on my personal and professional growth.

## The Fellowship Experience

The APSI—Dr. S Raja Sabapathy Leadership Award is a different kind of award. Most awards are given to honor someone's lifelong work or major contributions in a particular field. But the award started by the Association of Plastic Surgeons of India (APSI) in 2019 is different. It is meant to identify someone who shows promise as a future leader and support their growth in that direction. This award is not just an honor but also a challenge for the awardee—to live up to the trust and expectations that the association has placed in them. It also inspires recipients to stretch beyond their current boundaries, to reflect on what leadership truly means in their context, and to act with renewed intention.

One area that holds immense potential within our field is indigenous medical device innovation. In a country like India, where health care must reach millions with limited resources, affordable and locally developed solutions can have transformative impact. Imported devices often come at an unsustainable cost. For example, an imported intraocular lens costs nearly $100, while the same made by Aravind Eye Hospital is available for just $2. Similarly, imported heart valves are priced around $1,200, but the ones developed by Sree Chitra and TTK Healthcare are available at $400. A prosthetic leg imported from abroad may cost up to $15,000, whereas the Jaipur Foot is available for just $50, making mobility a reality for thousands. 2 These examples underline the importance of cost-effective, need-based, and scalable innovations tailored to local realities. Medical device innovation may not always offer every advanced feature, but it must meet essential requirements reliably and affordably. Despite the potential, to the best of my knowledge and search, very few plastic surgeons are currently involved in this space.

As a part of the fellowship initiative, and because of my core interest in medical device innovation, I visited Biomedical Engineering and Technology Incubation Centre (BETiC) at IIT Bombay. This indigenous medical device innovation facility was established in 2014 with the support of the S&T Commission of the Government of Maharashtra and the Department of Science & Technology of the Government of India. Since its inception in 2014 at the Indian Institute of Technology, Mumbai, BETiC has gathered over 400 unmet clinical needs from different hospitals. The team created 240 novel concepts, filed 55 patents, developed proof of concepts of 200 different medical devices in close collaboration with expert doctors. Further, they developed 25 devices, incubated 16 startups, and licensed 14 products for local industry partners. For medical device innovation, this is a giant accomplishment. The BETiC at IIT Bombay offered a unique ecosystem where interdisciplinary collaboration was fostering remarkable innovation. What intrigued me most was not their infrastructure, but the passion, and culture that powered their output.

My subsequent writings are a brief attempt to distil not only the observations I made there but also the insights I have gathered from what I have read, learned, and witnessed in the lives and work of the teachers and leaders in our fraternity. The lessons learned in that setting are not confined to that center alone—they resonate far beyond, embraced and applied globally by entrepreneurs and high-performing individuals alike.

Small teams can do remarkable things when everyone knows their role and takes responsibility. At BETiC, each person no matter how junior or senior was trusted to take ownership of their work. This trust encouraged innovative ideas and created a strong sense of shared purpose. It also demonstrated that leadership is not about giving direction—it is about helping others grow. Even when resources were limited, the team's energy and clear focus were inspiring.

One thing that stood out was how openly they accepted failure. When a prototype did not work, it was not hidden or ignored. It was treated as a lesson. This is so relevant to us in a clinical, academic, and research setting.

Most of all, BETiC's work culture—positive, focused, and welcoming showed that the environment we work in matters more than the tools we use. A good culture brings out the best in people.

Even before I visited BETiC, the idea behind the leadership award made me reflect on the impact of leadership in our day-to-day lives. I thought about the mentors who shaped my path—especially Dr. Yogesh Bhatt. If we reflect on the leaders and teachers and the influential people in our lives, I think all of us will realize that they have the same traits. And it is not about big speeches or fancy titles. It shows up in their small, everyday actions—choosing to listen, showing up with purpose, and being willing to adapt. It is their ability to bring people together. It is about the quiet strength they show, and how they help others rise without needing control. It is about how they listen, how they handle stress, and how they help others do better. These values matter just as much at home as they do at work. They affect how we handle conflict, make decisions, and stay grounded in uncertain times.

Leadership principles are not just tools for building teams or growing departments—they begin with the self. True leadership starts with self-awareness, discipline, and the courage to take responsibility for one's own growth. Before influencing others, we must learn to lead ourselves—with clarity, consistency, and purpose. These principles shape how we respond to challenges, make decisions, and navigate everyday life. In this way, leadership is as much about personal evolution as it is about professional progress.

This belief took shape in few personal projects. I had always wanted to do something beyond academics—to write something nonacademic, to teach, to create something meaningful. The idea of starting an online plastic surgery education platform too was lingering at the back of my mind. But there was always a quiet fear holding me back. What if I fail? Will anyone even find it useful? What will people think? The leadership award and my visit to BETiC acted as a catalyst. I finally acted on the ideas I had carried. I published *Navigating Dialysis – The AV Fistula Handbook for Patient Awareness* , which is being translated into multiple regional languages for patient education. The eBook on patient education in English and multiple other languages is available for free download at the following link https://www.plasticsurgeryeducation.com/courses/Navigating-Dialysis---The-AV-Fistula-Hand-book-67c3f57641458138eef63f41 . I started the online plastic surgery education platform for trainees and young plastic surgeons, and published a book titled *Cows Don't Give Milk and Other Short Stories* , available on Amazon.

One thing became clear though; you do not need to feel completely ready to begin. Leadership often means taking that first unsure step—showing up, doing the work, and learning as you go. The path reveals itself only when you start walking. The projects are still evolving and remain a continuous journey. However, the response so far has been humbling, deeply motivating, and genuinely inspiring. It is a reminder that sincere efforts, even when imperfect, can create meaningful impact, and usually finds its way to the people who need it.

At a professional level, I have been trying to implement these observations in the day-to-day functioning of my department. The results so far have been encouraging. I understand that meaningful change takes time, and the outcomes are often gradual and cumulative. I remain hopeful that my department will continue to grow and contribute in a meaningful way to the development of the fraternity.

## Reflections and Takeaways

- Leadership begins with taking responsibility of one's own growth.
- Leadership is a process, not a position.
- Empowered teams perform better than micromanaged ones.
- Learning from outside one's specialty expands capability and perspective.
- Failures are essential milestones on the path to innovation and growth.

## Conclusion

At a personal level, the APSI—Dr. S Raja Sabapathy Leadership Award has been a catalyst in reshaping my perspective on what leadership in plastic surgery truly means. For all of us—whether we are residents, faculty members, or practicing consultants—it is essential to engage with leadership not just as a position but as a daily practice. It is reflected in how we show up, the choices we make, and the consistency with which we strive for excellence, even when no one is watching.

In this spirit, I believe that medical device innovation holds immense promise, particularly in a country like India. Given our unique understanding of anatomy, function, esthetics, and patient needs, plastic surgeons are exceptionally well-placed to lead innovations that are both clinically relevant and socially impactful. From affordable external splints and wound care tools to novel prosthetics and reconstructive aids, the opportunities are vast and largely untapped.

The younger generation of plastic surgeons can consider venturing into the field of medical device innovation or any domain of research that holds transformative potential. With the deep understanding of form, function, and patient needs, plastic surgeons are uniquely positioned to identify gaps in clinical care and contribute meaningfully to the development of solutions. Leadership, ultimately, is about purpose and vision—beyond ourselves.
